# Nitric Oxide Has Differential Effects on Currents in Different Subsets of *Manduca sexta* Antennal Lobe Neurons

**DOI:** 10.1371/journal.pone.0042556

**Published:** 2012-08-03

**Authors:** Mark Higgins, Michael Miller, Alan Nighorn

**Affiliations:** Department of Neuroscience, University of Arizona, Tucson, Arizona, United States of America; Center for Genomic Regulation, Spain

## Abstract

Nitric oxide has been shown to regulate many biological systems including olfaction. In the moth olfactory system nitric oxide is produced in the antennal lobe in response to odor stimulation and has complex effects on the activity of both projection neurons and local interneurons. To examine the cell autonomous effects of nitric oxide on these cells, we used patch-clamp recording in conjunction with pharmacological manipulation of nitric oxide to test the hypothesis that nitric oxide differentially regulates the channel properties of these different antennal lobe neuron subsets. We found that nitric oxide caused increasing inward currents in a subset of projection neurons while the effects on local neurons were variable but consistent within identifiable morphological subtypes.

## Introduction

Experiments examining the localization of nitric oxide synthase (NOS) in the vertebrate brain using both NADPH diaphorase staining and NOS immunohistochemistry have demonstrated NOS-positive neurons throughout the brain [1], [2], [3]. Within the brain, high levels of staining were found in both the olfactory bulb and the olfactory epithelium suggesting that the nitric oxide (NO) signaling pathway is particularly important in the olfactory system [4]. Indeed, it has been suggested that the spheroidal neuropil that makes up the glomeruli of the olfactory bulb may make ideal structures in which to regulate the diffusion of NO [5]. In addition, the glial cells that surround each glomerulus may also serve as a boundary to limit interactions between glomeruli. Looking at other organisms, NOS has been found to be highly expressed in almost all olfactory systems, but the expression pattern of NO signaling components within the olfactory processing centers in the brain is quite variable. The exact role of NO-signaling in the processing of information within the olfactory bulb therefore remains unclear. NO could act to synchronize or modulate neural activity within a particular glomerulus without affecting surrounding glomeruli [5]. Because the axons of olfactory receptor cells that express a given olfactory receptor project to one glomerulus, or at most, a few glomeruli [6], [7], NO could be involved in coordinating, sensitizing, or modulating the olfactory system's response to a particular odorant. The NO signaling system also might mediate olfactory synaptic plasticity. Indeed, evidence for a form of olfactory learning mediated by NO has been found in mice [8], sheep [9], and honeybees [10] Recent studies have suggested that NO signaling is necessary for the efficient formation (but not recall) of olfactory memories [11], [12], [ and 13].

Our laboratory has used the moth *Manduca sexta*, as a model in which to investigate the function of NO signaling in the olfactory system. In *Manduca*, similar to other olfactory systems, olfactory receptor neurons project into a primary olfactory neuropil, called the antennal lobe (AL) in insects, where they synapse onto local interneurons (LNs) and projections neurons (PNs) within a glial cell ensheathed olfactory glomerulus. NOS in-situ hybridization and immunohistochemistry [14], [15] have demonstrated that NOS was located in the axons of the ORNs but was not present in either local or projection neurons within the AL. Soluble guanylyl cyclase (sGC), the best characterized target of NO, was not expressed within the olfactory receptor neurons or in most LNs but was highly expressed in a subset of PNs. Optical imaging methods have shown that NO is produced in response to odor stimulation in a glomerulus specific and concentration dependent manner [15]. The function of NO in the olfactory system has been more difficult to define. Using intracellular recording, we have shown that the manipulation of NO signaling has a profound effect on the overall excitability and odor responses of both LNs and PNs in the AL of *Manduca*. These responses are quite complex however with evidence of both sGC-dependent and sGC-independent effects. Some cells dramatically increased their excitability while others decreased their activity even within the same class of neurons [16]. Moreover, it was impossible to determine which effects were cell autonomous and which were due to changes in upstream elements of the circuitry.

In this paper we use patch clamp electrophysiology and cell culture to begin to examine the cell autonomous effects of NO on antennal lobe neurons. We found that NO consistently produced a dose dependent increase in the net inward currents of PNs. To ensure that these effects were not an artifact of cell culture, they were confirmed using in-vivo patch clamp recording. LN current responses to NO addition, however, were more complex and cell subtype specific. In one of the three subtypes of morphologically identifiable LNs, we observed not only two distinct basal current responses but also opposite responses to NO addition. In addition, three of the four subtypes showed significant differences in current response from control levels only at higher doses of NO. These results suggest that different subsets of AL neurons respond to NO differentially and that these effects are not all mediated by sGC, since some of the subsets do not express detectable levels of sGC [16].

## Methods

### Animals

*Manduca sexta* (*Lepidoptera*: *Sphingidae*) were reared on artificial diet in the laboratory from eggs and maintained at 25°C and 50–60% relative humidity under a long-day photoperiod regimen (17 hr light/7 hr dark).

### *In vitro* patch clamp

Neurons were derived from the dissociation and culturing of the AL according to previously established methods [17], [18], [19]. Briefly, brains were isolated from cold-anesthetized stage four (of the eighteen developmental stages) [20] metamorphosing adults with aseptic technique and transferred to sterile culture saline (supplemented-Leibovitz's L-15, Invitrogen). All solutions were adjusted to pH 7.0 and an osmolarity of 370–375 mOsm. Dissociated neurons were grown on glass cover slips in culture dishes for two weeks prior to patch clamping.

Just before recording, each cover slip was removed from its culture dish and placed in a recording chamber equipped with perfusion tubing. Culture medium was gradually replaced with recording saline (SIS: mM: 150 NaCl, 4 KCl, 2 CaCl_2_, 1 MgCl_2_, 10 HEPES buffer) before recording. Patch-clamp recordings were performed at room temperature using low resistance electrodes (5–10 MΩ) made from thin-walled borosilicate glass capillary tubes (TW150-3; World Precision Instruments, Sarasota, FL) pulled with a vertical puller (Type PP-83; Narishige, Japan) and filled with pipette solution (mM: 150 K aspartate, 8 NaCl, 2 MgCl_2_, 1 CaCl_2_, 11 EGTA, 2 ATP). The recording chambers for both *in vitro* and *in vivo* preparations were mounted on the fixed stage of an upright microscope (Olympus BX51WI, Center Valley, PA). A motorized four-axis controller (Siskiyou MC1100e, Grants Pass, OR) was mounted beside the microscope. The microscope was equipped with water-immersion objective lenses (Olympus UMPlan FL N, NA 0.30, and a LUMPlan FL N, NA 0.80).

Whole-cell currents were elicited in each cell by applying a series of 100 ms voltage steps in 10 mV increments from a holding potential of −70 mV (−90 to +50 mV), recorded with an Axopatch 200B amplifier, digitized on a Digidata 1322A, driven and analyzed with pClamp 10 software (Axon Instruments, Foster City, CA). Current signals were filtered with a low pass Bessel filter at 1 kHz. Leak currents were subtracted online using a p/4 protocol. Two runs per trial were averaged, and only the averaged traces were stored and analyzed. After baseline currents were established in SIS we applied the NO donor ProliNO (1-[2(carboxylato)pyrrolidin-1-yl]diazem-1-ium-1,2-dilate) (a generous gift from Dr. Katrina Miranda, Dept of Chemistry & Biochemistry, U of A) at 1 mM, 10 mM, 100 mM and 250 mM directly.

### *In vivo* patch clamp

Two to five day-old adults were dissected and prepared for whole-cell patch-clamp recordings by established procedures [21], [22], [23]. The head was removed from the body, the cuticle over the brain was removed, and the brain was exposed and rinsed with cold SIS (see above). The brain was then treated with 0.5 mg/ml collagenase (Sigma) in SIS for 20–30 min. in order to remove the glial cells that envelope the neuronal somata and then desheathed with fine forceps, transferred into a sylgard recording chamber (∼0.5 ml volume) and held in place using dissecting pins. A multibarrel glass perfusion electrode, placed just above a small rolled up piece of kimwipe the other end of which rested in the open head dorsal and anterior to the brain super fused the headspace continuously (1 ml/min) with SIS for about 15 min. before patching on to a cell.

After baseline currents were established in SIS we recorded current responses15 to 30 min. after the start of perfusion with the NOS inhibitor *O-*Nitro*-L-*arginine methyl ester (L-NAME; Sigma) dissolved in SIS and bath applied to the exposed brain at 1 ml/min. (at the minimal effective dose as determined by previous extracellular recording experiments 15 mM; Wilson et al. 2007). In addition, we recorded current responses to 10 mM and 30 mM L-NAME. Cells chosen for statistical analysis were those whose current profile remained consistent for at least 15 min. and returned to near baseline after washout of L-NAME.

### Data Analysis

Current-voltage relationships were obtained by measuring the peak amplitude of the current for each given membrane potential during the voltage step (at the end of the 100 ms pulse) and normalized to that cells current response to +50 mV in saline. Current traces from L-NAME/NO treated cells were subtracted from the recordings taken before the application of the drugs to reveal the current sensitive to the drug. All data are reported as means +/− standard deviation (SD) unless otherwise stated. Statistically significant differences in current were determined with ANOVA comparing mean current responses before (saline) and after drug (L-NAME) application or one way repeated measures ANOVA for the dose-responses to ProliNO both at the +50 mV level.

## Results

**Figure 1:**
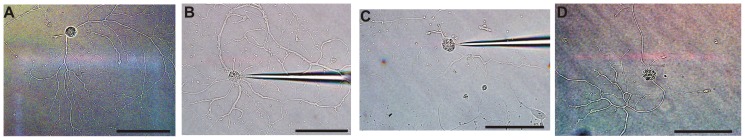
Cultured antennal lobe neurons examined in this study after 14 day *in vitro*. **A**. Projection neuron (PN) B. RickRack local interneuron (RR LN) showing recording electrode **C**. Fuzzy compact local interneuron (FC LN) D. Symmetrical local interneuron (SM LN). Scale bar = 50 µm.

To investigate the cell autonomous effects of NO on antennal lobe neurons, we took advantage of the robustness of *Manduca* AL neuron cell culture. In culture, *Manduca* AL neurons extend very healthy processes and can easily be characterized morphologically as being either PNs or LNs (figure 1). Moreover, there are also several distinct morphological subtypes of AL neurons [18], [19]. Using these cultures we examined the effects of precisely controlled amounts of NO on morphologically distinct subtypes of AL neurons by measuring their current responses to voltage steps before, during and after NO application. In addition we measured the current responses of PNs *in vivo* before, during and after NO inhibition in adults.

### In vitro whole-cell patch-clamp recording: NO has dose-dependent effects on a subset of PNs

**Figure 2:**
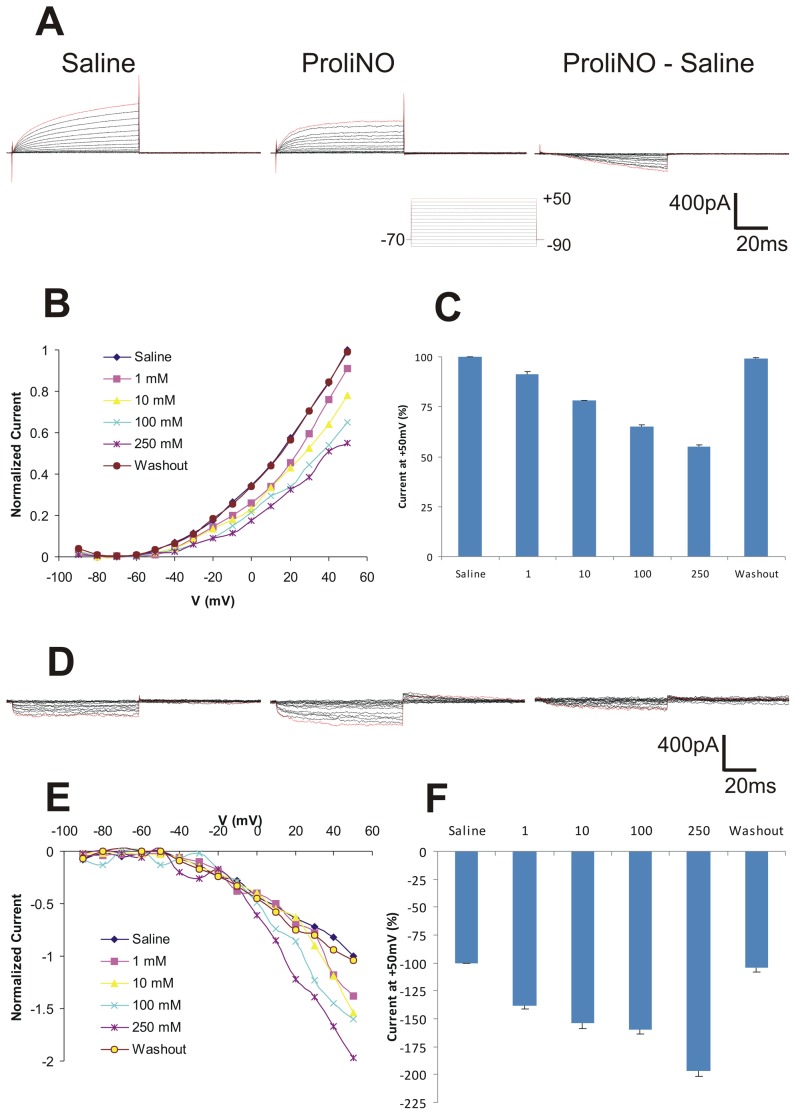
NO addition attenuates outward current of two subsets of PNs *in vitro*. **A**. Representative trace of PN with basal outward current *in vitro* showing (left to right) saline (control), ProliNO (100 mM) and subtracted current responses, elicited by the depolarization protocol (*bottom*): Voltage stepped from −90 to +50 mV in 10 mV increments for 100 ms from a holding potential of −70 mV. **B**. I–V plot of normalized current for control, increasing ProliNO doses (1, 10, 100, &250 mM), and washout (mean, n = 4). **C**. NO addition inhibited significantly the steady-state outward current of PNs with basal outward current in a dose-dependent manner (means +/− SD, n = 4). **D–F**. NO addition enhances significantly inward current responses of PNs with basal inward current. **D**. Representative traces of saline, ProliNO (100 mM) and subtracted current responses. **E, F**. Normalized I–V plot and histogram showing responses of *in vitro* PNs with basal inward current responses to increasing doses of ProliNO (Means +/− SD, n = 4).

We began by examining the effects of NO on PNs in culture (figure 1A). Cultured neurons do not have ORN input (the source of NO) and thus lack NO input. Moreover, the culture density was kept low so that the effects of NO on these neurons were cell autonomous. Even though they were morphologically identical the baseline responses of PNs fell into two categories. One subset showed a net inward current (ranging from −258 to −273 pA) and the other subset showed a net outward current (ranging from 798 to 815 pA) in response to the voltage steps (100 ms pulses of 10 mV steps from −90 to +50 mV with a −70 mV holding potential). To avoid missing data that might be lost in averaging the responses from these two very different subsets, we analyzed each subset separately. We determined the dose-response profile of these cells by recording the current responses in saline and then in increasing ProliNO concentrations (1, 10, 100 and 250 mM). Not all of the cells responded to the exogenously applied NO. Roughly 30% of the PNs that were examined responded to the changes in voltage but did not change those responses when NO was applied at any concentration. These cells were not included in the statistical analysis. A representative PN with a basal outward current showed a net inward deflection of current in response to NO addition (figure 2A). The normalized I–V plot (normalized to +50 mV saline current) of the current responses measured near the end of the 100 ms pulse showed activation at −50 mV and a decrease in net current corresponding with increasing amounts of NO (figure 2B). NO caused a significant, dose-dependent decrease in outward current in these PNs (figure 2C): 8.8+/−1.2% below control (saline) current in 1 mM; 22+/−0.2% in 10 mM; 34.8+/−0.7% in 100 mM; 44.8+/−0.6% in 250 mM (ANOVA, F = 847.1, p<0.0001, n = 4).

In the PNs exhibiting a basal inward current response we observed a significant, stepwise, dose-dependent increase in inward current with increasing NO concentration. The subtracted current trace of one of these PNs shows the net inward deflection of current in response to NO addition (figure 2D). As with all the PNs in this study these responded to increasing NO addition with increasing net inward current (figure 2 E, F): 38+/−3% below control (saline) current in 1 mM; 54+/−5.1% in 10 mM; 60+/−3.7% in 100 mM; 97+/−4.4% in 250 mM (ANOVA, F = 1015, p<0.0001, n = 4). Note that we describe these results as an increase in the inward current purely to be consistent with the direction of the basal currents. The ionic basis of the change is unknown and therefore could actually be the result of an increase in an inward current or a decrease in an outward current. We report here only the change in the net current.

### *In vitro* whole-cell patch-clamp recording: NO has cell-autonomous effects on local interneurons (LNs)

In order to investigate the effects of NO on the channel properties of LNs in isolation we performed whole-cell patch-clamp on cultured LNs perfused with SIS followed by increasing concentrations of exogenous NO (see M & M). *Manduca* LNs are categorized first by whether they have arborizations restricted to a few glomeruli versus those with arborizations in many glomeruli, as well as on their morphology. Cultured LNs, unlike PNs, all lack an axon-like process and have larger soma [18]. We identified three different morphological subtypes of LNs. Rickrack (RR) LNs (figure 1B), so-called because of their undulating array of multiple first and second order branches, are likely multiglomerular in vivo and have an irregular shaped soma. Fuzzy Compact (FC) LNs (figure 1C) also have an irregular shaped soma but have fewer and shorter branches than RR LNs, while Symmetrical (SM) LNs (figure 1D) have a relatively ovoid soma and several second-order branches coming off of a single branched first order arbor, suggesting uni-glomerularity [18]. As with the PNs, not all of the LNs responded to the exogenous NO. In this case 44% of RR, 47% of FC and 58% of SM LNs responded to NO and were included in the analyses.

**Figure 3:**
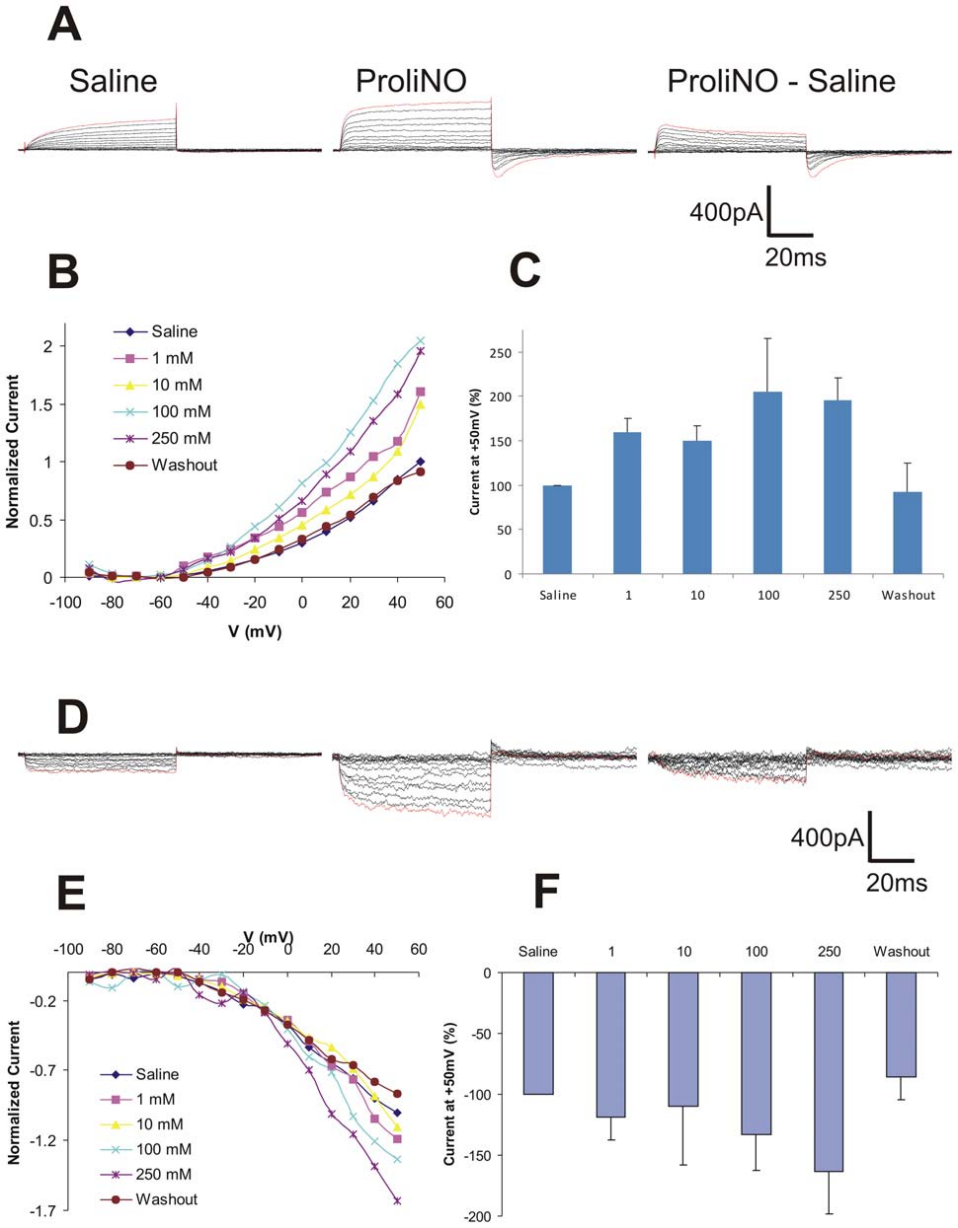
NO addition enhanced outward current of one subset of *in vitro* RR LNs and inward current of another subset. **A**. Representative traces showing (left to right) saline (control), ProliNO (100 mM) and subtracted current responses of an RR LN with basal outward current. **B**. I–V plot of normalized current responses of *in vitro* RR LNs in saline, increasing ProliNO doses, and washout (means, n = 3). **C**. NO addition significantly enhances an outward current in RR LNs with basal outward current in 1 mM,10 mM, and 250 mM ProliNO (means +/− SD, n = 3). **D–F**. Same as above for RR LNs with basal inward currents. F. NO addition significantly enhances inward currents of RR LNs with basal inward current at 250 mM ProliNO (means +/− SD, n = 4).

RR LNs, like PNs, demonstrated two subtypes of whole-cell basal current responses – those with a net inward current and those with outward current. A representative trace of a RR LN with basal outward current shows a rapidly activating outward current followed by a slowly inactivating inward current in response to NO (figure 3A). A dose response I–V plot (normalized to the saline current response at +50 mV) shows activation at −50 mV and the increasing outward current responses to increasing NO (figure 3B). In these cells we observed a significant outward current deflection in response to NO addition (one way repeated measures ANOVA, F = 23, p<0.01; mean +/− SD; n = 3) (figure 3C).

RR LNs with basal inward current responded with increasing inward deflecting current with NO addition (figure 3D–F). A representative trace shows that this subset of RR LNs, unlike the other subset of RR LNs, responded to NO addition with a slowly activating inward current (figure 3D). A normalized dose-series I–V plot shows activation at −50 mV to −40 mV and the increasing inward current responses to increasing NO addition (figure 3E). This subset of RR LNs showed significant inward current deflection in response to NO addition (one way repeated measures ANOVA, F = 18, p<0.01; mean +/− SD; n = 4) (figure 3F).

**Figure 4:**
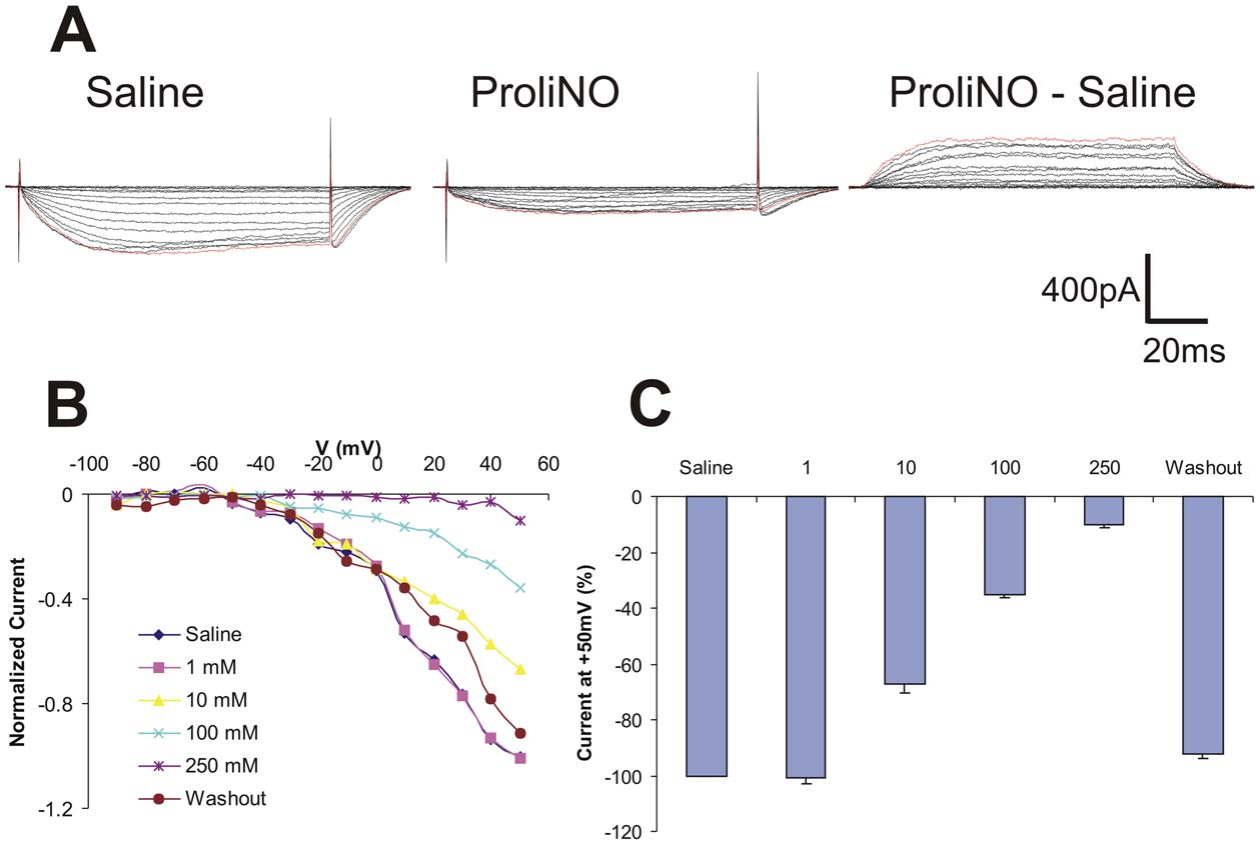
NO addition enhances outward current of FC LNs *in vitro*. **A**. Representative traces showing (left to right) saline (control), ProliNO (250 mM) and subtracted current responses. **B**. I–V plot of normalized current responses of *in vitro* FC LNs in saline, increasing ProliNO doses, and washout (means, n = 4). **C**. NO addition significantly enhances an outward current in a dose-dependent manner above 1 mM ProliNO.

FC LNs exhibited a basal inward current and responded to NO addition with decreasing inward current (figure 4). A representative trace shows, unlike any of the other LNs with basal inward current that we observed, a net outward current in response to NO (250 mM) (figure 4A). A normalized I–V plot shows activation at about −50 mV and attenuated inward current responses to NO (figure 4B, mean, n = 4). FC LNs appear unique among the LNs that we observed, responding to NO application (above 1 mM) with significant, stepwise, dose-dependent net reversal of inward current above control (one way repeated measures ANOVA, F = 1849, p<0.01; mean +/− SD; n = 4) (figure 4C).

**Figure 5:**
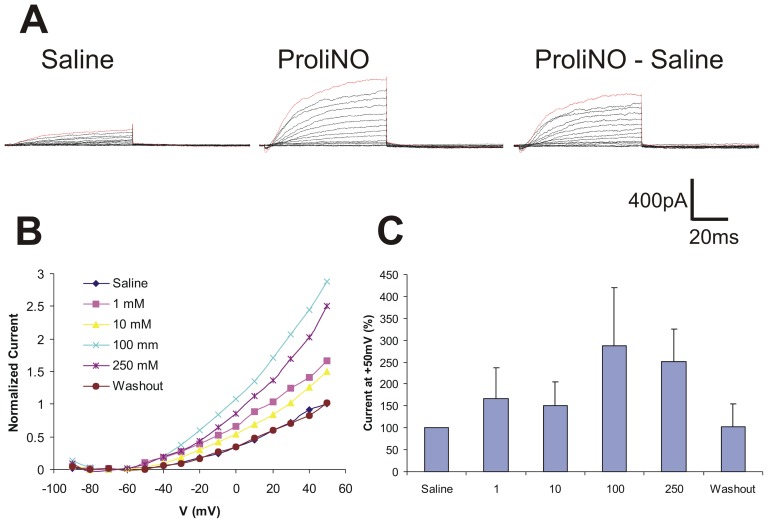
NO addition enhances outward currents of SM LNs *in vitro* at higher doses. **A**. Representative traces showing (left to right) saline (control), ProliNO (100 mM) and subtracted current responses. **B**. I–V plot of normalized current responses of *in vitro* SM LNs in saline, increasing ProliNO doses, and washout (means, n = 4). **C**. NO addition significantly enhances an outward current at 100 & 250 mM ProliNO.

SM LNs in saline responded to depolarizing voltage steps with outward current. A representative trace shows a very small, transient inward current followed by a rapidly activating outward current in response to NO (100 mM; figure 5A, *right*). Higher concentrations of NO increased outward current in SM LNs with subsequent washout (figure 5B, C). A normalized I–V plot shows activation at −50 mV and outward current responses to NO (figure 5B, mean, n = 4). NO addition resulted in significant outward current deflection above control levels (one way repeated measures ANOVA, F = 23, p<0.01; mean +/− SD; n = 4) (figure 5C).

### *In vivo* whole cell patch clamp recording: NO inhibition enhances a steady-state outward current

**Figure 6:**
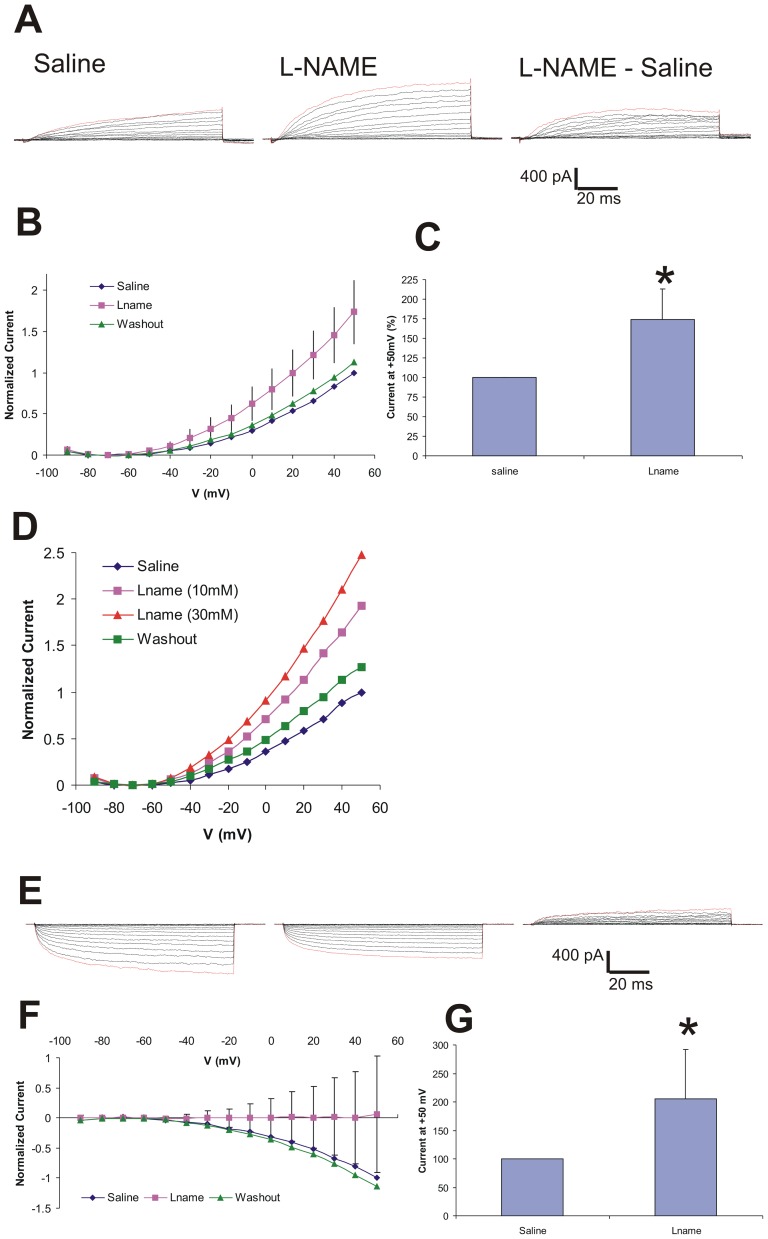
NO inhibition (L-NAME) *in viv*o results in increasing outward current in some PNs (A, B, C) and decreasing inward current in others (D, E, F). **A**. Representative traces of net outward currents elicited by the depolarization protocol (see figure 1) before (saline), 30 min. after treatment with L-NAME, and the difference (L-NAME - Saline). **B**. Normalized current I–V plot for control, L-NAME (15 mM), and washout (mean +/− SE; n = 4). **C**. L-NAME significantly increased an outward current (p<0.05). **D**. I–V plot showing increasing doses on NO inhibitor (L-NAME) *in vivo* results in dose-dependent increases in outward current. **E–G**. Current responses of neurons with inward basal response (mean +/− SE, n = 5). L-NAME decreases inward current significantly (p<0.01).

The PNs that we recorded from in culture were never exposed to NO prior to the experiment. To ensure that the effects that we saw were not due to culture conditions, we patched onto cell bodies in the medial cell body cluster (all of which are PNs) in the *Manduca* AL and measured their baseline responses. As with PNs in culture we identified two distinct PN subtypes *in vivo*: PNs with basal outward current responses (figure 6A–D) and PNs with basal inward current responses (figure 6E–G), both of which activated at −50 mV (figure 6). Since these neurons were already in the presence of baseline levels of NO in the AL, we examined the effects of NOS inhibition. In both subtypes of PNs, exposure to 15 mM L-NAME resulted in a net increase in the outward current. All of the cells examined showed a response to the drug.

A representative trace of a PN with basal outward current shows that L-NAME increased the net outward current and that the affected current is slowly activating and appears to be deactivating near the end of the 100 ms pulse (figure 6A). A normalized I–V plot of this subgroup of PNs shows the increased outward current in response to L-NAME (figure 6B). The average non-normalized current increased from 496 pA in saline to 807 pA in response to L-NAME resulting in significant outward current responses of 74+/−39% over control levels (paired Student's t-test, p≤0.05, n = 4) (figure 6C). To confirm these results we examined the effects of higher and lower concentrations of L-NAME (10 and 30 mM). We found dose-dependent increases in outward current in these PNs. An I-V plot shows that 10 mM L-NAME resulted in a 92% increase (883 pA); while 30 mM L-NAME increased the current 147% (1136 pA) above control (saline) levels (459 pA) (figure 6D). PNs with basal inward current responses responded with a similar slowly activating current in response to L-NAME (figure 6E). The normalized I–V plot of these cells shows that the mean current response to L-NAME is an outward deflection (figure 6F). In this PN subset L-NAME resulted in an average non-normalized current increase to −142 pA, from −422 pA in saline. This was a significant response to L-NAME by these PNs, increasing their outward current 106+/−86% over control (saline) levels (p≤0.01, n = 5) (figure 6G).

## Discussion

Nitric oxide synthase is highly expressed in the primary olfactory neuropil of almost all animals and is likely to play an important role in the processing of olfactory information. To further understand the effects of NO on olfactory neurons we used whole cell patch clamp electrophysiology to examine the cell autonomous effects of NO on *Manduca sexta* antennal lobe (AL) neurons. The fact that many a sizable percentage of neurons did not respond to NO was surprising and the significance of this finding is unknown. Most PNs (∼85%) express sGC and most LNs (∼90%) do not. Even though more PNs than LNs responded to NO (70% compared to 52%) the percentages did not match the expression of sGC. This, combined with the variable responses of the cells that did respond to NO, suggested that the explanation is more complicated and further studies examining the expression of affected ion channels is warranted.

Our results show that NO has cell autonomous modulatory effects on the currents of a significant subset of LNs in the *Manduca* AL (figures 3–5). The morphological and physiological diversity of LNs in the insect AL is well documented [17], [18], [24], [25]. Our results expand on that diversity as we show *in vitro* that even within one morphological category (RR LNs) NO addition increases the net outward current in one subset and increases the net inward current in another (figure 3). Of the two other LN cell types studied here, FC LNs exhibited dose-dependent decreasing inward current in response to NO addition, while SM LNs responded with increasing outward current only at higher concentrations of NO (figures 4 & 5, respectively).

The variable responses of the different LNs to NO addition suggest the existence of different mechanisms of NO action. This might allow for a greater degree of fine tuning in the odor processing circuitry. In rat striatal neurons, for example, different cell subtypes responded differently to NO and sGC inhibition [26]. One possible mechanism accounting for variable responses may be the existence of different NO targets in the different LNs. Most LNs do not express detectable levels of sGC [15] suggesting the effects that are seen here are likely to be mediated by direct S-nitrosylation [27], [28], [29]. It has been suggested that this pathway may require higher concentrations of NO [30], [31] and may proceed with slower kinetics than cGMP-mediated signaling cascades [32], [33], [34]. This is consistent with what we observed in some of the LN subtypes.

There could also be different channels expressed, as well as different numbers of the same channels expressed, on the different LN sub-types. For instance, the two subtypes of RR LNs could be expressing outward and inward rectifying K+ channels, respectively. In the substantia nigra voltage-activated sodium channels are found at different densities on GABA versus dopamine projection neurons [35]. Other cases of NO addition leading to increased inward current in inhibitory neurons have been found, for instance patch-clamping of rat thalamus GABAergic TRN neurons showed significant inward current deflection in response to NO donors [36].

We observed two distinct current response populations of PNs, one with basal outward current (figure 2A–C, figure 6A–D) and the other with basal inward current (figure 2D–F, figure 6E–G) suggesting the existence of PN subtypes. And while there are five known PN subtypes based on morphology and whole-cell current characteristics [17] with probably more functional classes based on spiking behaviors allowing for the processing of the various pheromonal and non-pheromonal odor stimuli [37] the PNs that we analyzed were not representative of all the subtypes. The PN response to NO inhibition/addition, however, was consistent: both subtypes responded with net outward current with NO inhibition (figure 6) and net inward current with NO addition (figure 2). This suggests that while there are different PN subtypes (expressing different channels, etc.) NO may function by increasing the effectiveness of excitatory inputs in PNs in *Manduca*. NO inhibition of K^+^ currents, for example, has been seen in other invertebrates including snail buccal neurons [38], [39] and *Aplysia* MCC neurons [40] which are depolarized by exogenous NO by inhibiting K^+^ currents. This is similar to the effects of exogenous NO on the neurons of some vertebrates, including pituitary GH3 cells [41] and in mouse pancreatic B cells, where NO inhibition via L-NAME led to depolarizing currents by inhibiting K^+^ channels [42]. On the other hand, in mouse MNTB and hippocampal C3 cells NO regulates postsynaptic excitability through both inhibition (Kv3) and augmentation (Kv2) of K^+^ currents [43].

PNs *in vitro* were depolarized by NO addition and, consistent with this result, PNs *in vivo* were hyperpolarized by NO inhibition. This suggests that the resting activity of ORN axons results in tonic levels of NO in every glomerulus. This low level of NO could be priming the PNs to respond once local levels of NO have increased in the specific odor stimulated glomerulus [15]. PNs taken from the brains of stage 4 animals and cultured for two weeks exhibit morphological characteristics, as well as current responses, similar to those seen in the same populations of antennal lobe neurons in the adult [17], [22].

The variable effects of NO on LNs and the consistent effect of increasing inward currents, and thus, increasing the sensitivity of, PNs suggests that NO plays a crucial regulatory role in olfactory processing by modulating a variety of currents in the central neurons that make up the circuitry of the antennal lobe. The differential affects of NO on these various cell type (and subtype) membrane properties points up the complexity of the NO pathway in the olfactory system. As in other systems, both vertebrate [26], [44], [45], [46] and invertebrate [38], [47], NO regulates olfactory processing in *Manduca* by affecting different AL neuron subtypes which likely express different channels as well as different NO sensitive pathways.
